# Conscious versus unconscious thinking in the medical domain: the deliberation-without-attention effect examined

**DOI:** 10.1007/s40037-014-0126-z

**Published:** 2014-06-04

**Authors:** Benno Bonke, Robert Zietse, Geoff Norman, Henk G. Schmidt, Roger Bindels, Sílvia Mamede, Remy Rikers

**Affiliations:** 1 Unit of Medical Psychology & Psychotherapy, Department of Psychiatry, Erasmus University Medical Center, PO Box 2040, 3000 CA Rotterdam, the Netherlands; 2 Department of Internal Medicine, Nephrology, Erasmus University Medical Center, PO Box 2040, 3000 CA Rotterdam, the Netherlands; 3 Program for Educational Research and Development, McMaster University, Hamilton, ON Canada; 4 Department of Psychology, Social Sciences, Erasmus University, PO Box 1738, 3000 DR Rotterdam, the Netherlands

**Keywords:** Unconscious thought, Clinical reasoning, Expertise

## Abstract

Previous studies have shown that with important decisions, unconscious thought has surprisingly led to better choices than conscious thought. The present study challenges this so-called ‘deliberation-without-attention effect’ in the medical domain. In a computerized study, physicians and medical students were asked, after either conscious or unconscious thought, to estimate the 5-year survival probabilities of four fictitious patients with varying medical characteristics. We assumed that experienced physicians would outperform students as a result of their superior knowledge. The central question was whether unconscious thought in this task would lead to better performance in experts or novices, in line with the deliberation-without-attention effect. We created four fictitious male 60-year-old patients, each of whom with signs and symptoms related to likely prognosis, from 12 (Complex) or 4 (Simple) categories. This manipulation resulted in objectively different life expectancies for these patients. Participants (86 experienced physicians and 57 medical students) were randomly allocated to the Simple or Complex condition. Statements were randomly presented for 8 s. Next, each participant assessed the life expectancies after either conscious or unconscious thought. As expected, experienced physicians were better in assessing life expectancies than medical students. No significant differences were found in performance between conscious and unconscious thought, nor did we detect a significant interaction between expertise level and mode of thought. In a medical decision task, unconscious thought did not lead to better performance of experienced physicians or medical students than conscious thought. Our findings do not support the deliberation-without-attention effect.

## Introduction

Medical knowledge, intelligence, and reflective reasoning [1, 2] are considered to be the cornerstones of a doctor’s daily work. Yet where critical decisions are to be taken, many doctors are aware of the importance of ‘gut feelings’ and ‘educated intuition’ [3, 4], i.e. unconscious reasoning that may guide behaviour more than conscious knowledge does [5–8]. It is important to distinguish two ways in which the notion of ‘unconscious’ processes has arisen in the literature. The first is a contrast with conscious analytical processing as a component of a dual process model of reasoning [9] and is synonymous with non-analytical or so-called System 1 thinking. The ‘gut feeling’ alluded to above likely reflects this process, which occurs concurrent with or even ahead of analytical reasoning. A very different kind of unconscious thought arises from studies of the deliberation without attention (DWA) effect, wherein individuals may even profit from unconscious thought [10]—i.e. ‘active thought processes that take place while attention is directed elsewhere’ [11] occurring hours or even days after the event—and take better decisions than after conscious thought. [12–17] This effect [12] has, however, been challenged [18–25] (see Acker [26] for a meta-analysis and Baumeister et al. [27] for a review) and the findings are not uniformly supportive. In particular, in the medical domain, Mamede et al. [24] recently found that conscious thought resulted in better decisions with complex tasks and experts, contradicting the DWA effect, although it provided no advantage over a DWA condition with students. It is not clear why the effect of DWA would have different effects at different levels of expertise. Conversely, De Vries et al. [15] reported more correct diagnoses of psychiatric case vignettes after unconscious processing than after conscious processing.

Studies showing the DWA effect [12–17] have mainly focused on decisions that were of importance to the decision maker, such as ‘what is the best car to buy?’ (although often in hypothetical experimental situations). But, does the effect also occur when decisions are made that are important for others, which is daily practice for doctors? In particular, decisions related to prognosis and therapy need careful consideration, and this is exactly what one may expect from experienced physicians. If, in either Simple or Complex situations, delaying a decision to allow time for unconscious processes to intervene is superior to conscious deliberation, physicians would be advised to postpone a decision and ‘sleep on it.’ This would in many ways be at variance with common sense and daily practice. The claims that originate in studies that supported the DWA effect have, however, attained a large audience [e.g. 14–17, 28, 29], so we decided to test if these claims hold true for individuals who make far-reaching decisions daily, i.e. physicians. Likewise, if a DWA effect is present in clinical doctors, the medical educational system must incorporate its repercussions.

In the present study, we confronted physicians and medical students with fictitious patients having objectively determined different life expectancies. Medically relevant characteristics were provided in either a Simple or a Complex task, closely following the design of Dijksterhuis et al. [12]. We aimed to simulate clinical practice where doctors receive a host of information for their patients and then must decide on whom to take action most urgently. Our participants assessed the patients’ life expectancies after either conscious or unconscious thought. We included level of expertise because it is at the basis of optimal decision-making and has been largely ignored in most previous DWA studies. Given our previous findings [24] we expected that experienced physicians, as a result of their large knowledge base built up over the years, are not affected in their performance by mode of thought. In particular, we were interested to find out if a DWA effect occurred in the complex task, because this most closely resembled the task where positive effects had been demonstrated [12].

## Methods

### Participants

We recruited 150 participants from the departments of internal medicine of academic and non-academic hospitals and from a university medical centre in the Netherlands between April 2009 and May 2011. They included consultants and residents in internal medicine (experts) and advanced medical students starting their clerkships (novices). Experts differed substantially in experience. The data of six participants with inconsistent response sheets and of one participant with language problems were removed. The remaining participants were 86 experts and 57 novices.

### Design and materials

The study was approved by the Ethics Committee at the Department of Psychology, Erasmus University Rotterdam, the Netherlands. We created stimulus materials (i.e. cases) by attributing medically relevant characteristics to four fictitious male patients, phrased in statements. We used 12 signs and symptoms that are known to affect a patient’s life expectancy, such as blood pressure, serum creatinine, body mass index (BMI), and alcohol use. Common Dutch surnames (e.g. *Klaassens*) were used for the patients, renamed here as *Lo*, *Med1*, *Med2*, and *Hi* (see below). Statements were randomly displayed on a computer screen and each statement clearly identified the patient to whom the information belonged, e.g.* Mr Klaassens drinks 4–5 units of alcohol daily*. If a statement included a numerical value (e.g. from lab tests) a qualification was added, i.e. *elevated, lowered,* or *normal* (or *obesity* for BMIs >30), as follows: Mr Jansen´s serum creatinine is 70 μmol/L (normal) or Mr De Vries’s blood pressure is 170/110 mmHg (elevated). We attributed neutral (or favourable) and unfavourable statements to the patients in such a way that they differed in the number of neutral/favourable and unfavourable characteristics. Patient *Lo* was intended to have the lowest life expectancy with 25 % neutral/favourable and 75 % unfavourable characteristics, patients *Med1* and *Med2* intermediate with both 50 % neutral/favourable and 50 % unfavourable, and patient *Hi* the highest life expectancy with 75 % neutral/favourable and 25 % unfavourable characteristics. Also, we defined a subset of four characteristics for each patient, with the same distribution of neutral/favourable and unfavourable characteristics, to be used in the simple versions of the study (Table 1). The effectiveness of our manipulation was confirmed by two board-certified experts in internal medicine who independently studied the characteristics of our fictitious patients. Both considered these patients to be typical for what one might expect in an outpatient clinic of internal medicine. They rated the life expectancy of patient *Lo* as the lowest, that of patient *Hi* as the highest, and that of patients *Med1* and *Med2* in between. We used psychological software (E-Prime 2.0 Professional: www.scienceplus.nl) to instruct our participants and present the statements and anagram task (see below) on a laptop computer.

**Table 1:** Medical characteristics as distributed over the four patients; each characteristic was neutral or favourable (+) in two patients and unfavourable (−/−) in the other two

Medical characteristic^a^	Patient *Lo*	Patient *Med1*	Patient *Med2*	Patient *Hi*
*Smoking*	−/−	+	−/−	+
*Body mass index* (kg/m^2^)	+ (22)	−/− (30)↑	+ (23.5)	−/− (30.5)↑
*Family history*	−/−	+	−/−	+
*Total cholesterol* *(mmol/L)*	−/− (7.3)↑	−/− (7.2)↑	+ (5.3)	+ (5.1)
Blood pressure (mm/Hg)	−/− (170/110)↑	+ (140/80)	−/− (180/105)↑	+ (135/85)
Alcohol use	−/−	+	−/−	+
Haemoglobin (mmol/L)	−/− (6.3)↓	−/− (6.0)↓	+ (8.9)	+ (9.2)
Proteinuria (g/days)	−/− (1.3)↑	−/− (1.0)↑	+	+
Fasting blood sugar (mmol/L)	−/− (10.5)↑	−/− (11)↑	+ (5.0)	+ (4.5)
Body exercise	+	+	−/−	−/−
C-reactive protein (mg/L)	+ (3)	−/− (22)↑	+ (2)	−/− (23)↑
Serum creatinine (μmol/L)	−/− (125)↑	+ (70)	−/− (127)↑	+ (72)
	25 + 75 % −/−	50 + 50 % −/−	50 + 50 % −/−	75 + 25 % −/−

^a^Only the first four characteristics (in italics) were used in the Simple versions; ↑ = elevated (or obesity in case of BMI); ↓ = lowered

We devised an anagram task with randomly presented anagrams of the names of Dutch towns to divert attention in the unconscious thinking mode. We programmed four equivalent versions of our study, i.e. a Simple (SIM) and a Complex (CPX) version, each with both a Conscious (CSC) and an Unconscious (UNC) thinking task after presentation of the statements. Table 2 presents the study design.

**Table 2:** Study design

Mode of thought	Simple versions (SIM)	Complex versions (CPX)
Conscious (CSC)	4 × 4 statements	4 × 12 statements
	4 min conscious thought	4 min conscious thought
Unconscious (UNC)	4× 4 statements	4× 12 statements
	4 min anagrams	4 min anagrams

### Procedure

Participants volunteered and consented to take part in what was announced as an anonymous, computerized study into memory processes. They were seated in front of a laptop computer and informed that they would be presented, consecutively and in random order, with statements pertaining to four fictitious patients regarding relevant signs and symptoms obtained through history taking, physical examination, or laboratory findings. All patients were supposedly 60-year-old males visiting the outpatient internal medicine clinic, referred by their general practitioners with vague complaints. We randomly assigned participants to one of the four study versions and informed them that after presentation of the statements they were to assess the patients’ life expectancies. We urged them to pay close attention to each statement and to remember which statement belonged to which patient, with a view to assessing the life expectancies, without taking notes. Next, we showed three examples of statements and invited them to ask any questions about the procedure. Then they received, in random order, 16 (SIM-CSC and SIM-UNC) or 48 (CPX-CSC and CPX-UNC) statements—i.e. 4 or 12 statements × 4 patients—each for a duration of 8 s. After presentation of the statements, participants in the CSC versions were prompted to ‘think carefully about the life expectancies of the four patients for a few minutes’ and those in the UNC versions did the anagram task instead. After 4 min in all versions, participants received a response sheet to assess the patients’ life expectancies by writing down the names of the two patients who—in their opinion—had the most and the least favourable life expectancies. Next, they filled in, for each patient separately, their estimated probability that he would be alive in 5 years’ time, as follows: *I estimate the probability that Mr De Boer will be alive in 5* *years’ time to be …%*. The order in which the names appeared on the sheet varied between participants to prevent order effects. Participants stated the number of years they had had experience in internal medicine (none, ‘a few months or less’, 6–12 months, 1–5, >5 years). No further biographical data were obtained. All participants carried out their tasks individually, without being disturbed, and received a small token of appreciation for their participation.

### Data reduction

For each participant we ranked the percentages given to the four patients as follows. Rank 1 for the patient who had been given the lowest percentage, rank 4 for the one with the highest percentage given. In case of equal percentages, ranks were averaged. We then computed the rank correlation (Rho) between participant ranks and objective ranks (*Lo*: rank 1, *Med1* and *Med2:* both rank 2.5, *Hi*: rank 4) as our dependent variable (range −1.0 to +1.0). Thus Rho = −1.0 if a participant´s assessments ranked completely incorrectly; Rho = +1.0 if a participant ranked correctly.

### Statistical analysis

We analyzed differences in *Rho* for experts and novices using a 2 × 2 × 2 ANOVA with *Expertise level* (physician, medical student), *Mode of thought* (CSC, UNC), and *Condition* (Simple, Complex) as between-factors and *Rho* as the dependent variable. To generate sufficient statistical power (following Dijksterhuis et al. [12], Studies 1 and 2) we aimed at groups of at least 15 participants in the various conditions. All data were analyzed using SPSS version 18.

## Results

For the 57 novices, years of experience was ‘none’ for 55, and ‘a few months or less’ for 2 (one Simple/UNC, one Complex/UNC). For the 86 experts, i.e. residents or consultants with at least some experience, the levels of experience (‘a few months or less’, 6–12 months, 1–5, >5 years) were distributed as follows over the four conditions: Simple/CSC (*N* = 1, 3, 5, 5, respectively), Simple/UNC (*N* = 1, 2, 7, 3, respectively), Complex/CSC (*N* = 1, 2, 13, 12, respectively), Complex/UNC (*N* = 1, 2, 17, 11, respectively).

As a check upon our manipulation in the Simple and Complex versions, we compared mean ranks for percentages given for patients *Lo*, *Med1*, *Med2*, and *Hi*, respectively. With minor exceptions for novices in both Complex conditions and for experts in the Simple/UNC condition, mean ranks were in line with our manipulation: lowest for patient *Lo*, highest for patient *Hi*, and in between for the two other patients. We considered these results to be confirmation that our manipulation had created fictitious patients that clearly differed in life expectancy in the intended direction (Table 3).

**Table 3:** Mean ranks of percentages given by novices and experts in their assessments of life expectancy, after conscious or unconscious thought (lowest percentage: rank = 1; highest percentage: rank = 4)

Level of expertise	Simple/Complex	Conscious/Unconscious	Patient^a^	Mean	SE
Novices (*N* = 57)	Simple (*N* = 29)	CSC (*N* = 15)	Lo	2.03	0.27
			Med1	2.40	0.25
			Med2	2.37	0.24
			Hi	3.20	0.29
		UNC (*N* = 14)	Lo	2.14	0.28
			Med1	2.57	0.25
			Med2	2.57	0.25
			Hi	2.71	0.30
	Complex (*N* = 28)	CSC (*N* = 15)	Lo	2.43	0.27
			Med1	2.47	0.25
			Med2	2.20	0.24
			Hi	2.90	0.29
		UNC (*N* = 13)	Lo	1.54	0.29
			Med1	2.89	0.26
			Med2	2.81	0.26
			Hi	2.77	0.31
Experts^b^ (*N* = 86)	Simple (*N* = 27)	CSC (*N* = 14)	Lo	2.18	0.28
			Med1	2.25	0.25
			Med2	2.46	0.25
			Hi	3.11	0.30
		UNC (*N* = 13)	Lo	1.96	0.29
			Med1	1.92	0.26
			Med2	3.12	0.26
			Hi	3.00	0.31
	Complex (*N* = 59)	CSC (*N* = 28)	Lo	1.68	0.20
			Med1	2.54	0.18
			Med2	2.41	0.18
			Hi	3.38	0.21
		UNC (*N* = 31)	Lo	1.98	0.19
			Med1	2.24	0.17
			Med2	2.52	0.17
			Hi	3.26	0.20

*CSC* conscious thought, *UNC* unconscious thought, *SE* standard error

^a^Patient *Lo* has the lowest life expectancy, patient *Hi* the highest, and patients *Med1* and *Med2* in between; the lower the mean rank for Patient *Lo* and the higher the mean rank for Patient *Hi*, the more accurate the assessments

^b^We recruited a greater number of experts to participate in the complex conditions to examine differences in level of expertise; there was no significant difference, therefore we collapsed the two groups

In an initial analysis of the Rho coefficients, the difference in mean scores between experts and novices (Expert, *M* = 0.44, Novice, *M* = 0.26) did not quite reach statistical significance (*F*(1,142) = 3.20, *p* = 0.08). However, a simplified analysis omitting *Mode of thought* and *Condition* did result in a significant difference (*p* = 0.04). We found no significant main or interaction effects for any of the other factors in the ANOVA (*Mode of thought*: *F*(1,142) = 0.23, *p* = 0.64; *Condition*: *F*(1,142) = 0.15, *p* = 0.70; interactions: all *p*s > 0.1). When focusing on the DWA effect, we did not find a significant main effect of DWA (*F*(1,142) = 0.23, *p* = 0.64 for all cases, *F*(1,86) = 0.19, *p* = 0.67 for complex cases). Average rank correlations for all conditions are presented in Table 4.

**Table 4:** Mean rank correlations (Rho) between participant ranks and objective ranks

Simple/Complex	Conscious/Unconscious	Mean	SE
Novices			
Simple	CSC	0.35	0.15
	UNC	0.19	0.16
Complex	CSC	0.12	0.15
	UNC	0.37	0.16
Experts			
Simple	CSC	0.31	0.16
	UNC	0.47	0.16
Complex	CSC	0.53	0.11
	UNC	0.47	0.11

*CSC* conscious thought, *UNC* unconscious thought; higher means correspond with better task performance

## Discussion

The present study investigated task performance in medical doctors and students in evaluating fictitious patients with different life expectancies. Medically relevant characteristics were provided in either a Simple or a Complex task, closely following the design of Dijksterhuis et al. [12]. We expected doctors not to be influenced by mode of thought (i.e. conscious vs. unconscious). As predicted, the deliberation-without-attention effect was absent. Contrary to what this effect predicts, task performance after unconscious thought was not better than after conscious thought [12–17].

We verified that our fictitious patients indeed differed in the assumed direction: both the objective statements by the two board-certified internists and the mean ranks over all participants (Table 3) supported our intended manipulation. Compared with Studies 1 and 2 by Dijksterhuis et al. [12], in which such verification was lacking, our double manipulation check can be considered a strength of our study. Also, our participants had a specific expertise in the domain in which they were tested and the chosen age and gender of our fictitious patients made assessments of the life expectancies relevant. Lastly, we carefully selected characteristics that were objectively relevant for a patient’s life expectancy, which resulted in combinations of characteristics that could go together well in real patients.

It would be interesting to see whether a DWA effect occurs when medical decisions are directly important for decision-makers themselves. On the other hand, the present study reaffirms previous research showing that expert doctors do not benefit from unconscious thought while making decisions in complex situations. [24].

The important lesson that can be learned from the present study, and one that opposes the central claim of the DWA framework, is that there is no reason to assume that practising physicians do well to distract themselves with other tasks before making important decisions for patients.

A possible weakness in our study is the artificiality of the experimental setup. Doctors in clinical practice who receive relevant patient information and medical characteristics seldom have to rely on their memory only before deciding what action to take.

## Conclusion

In a relevant medical decision task (Simple or Complex), unconscious thought did not lead to better performance than conscious thought. Our findings seem to indicate that the DWA effect, if theoretically valid, does not take place in physicians or in medical students. Our decision task, although theoretically similar to previous studies, failed to reveal a superior effect of unconscious thinking.

## Essentials

- The basic design for the deliberation-without-attention effect was successfully extrapolated to a decision task in the medical domain, where doctors and medical students assessed the life expectancies of virtual patients as a computerized medical decision task.
- The deliberation-without-attention effect could not be replicated.
- Unconscious thought did not lead to better performance of experienced physicians or medical students than conscious thought.
- Experts made more accurate assessments of life expectancies than novices.
